# Assessment of knowledge and attitude of pregnant women towards antenatal ultrasound in University of Gondar comprehensive specialized hospital at antenatal care clinic, Northwest Ethiopia

**DOI:** 10.1371/journal.pone.0292496

**Published:** 2023-11-08

**Authors:** Belayneh Mengistie, Sileshi Ayele, Winta Tsehaye, Atsede Mazengia, Maereg Wolde

**Affiliations:** 1 Department of Obstetrics and Gynecology, School of Medicine, College of Medicine and Health Sciences, Debre Markos University, Debre Markos, Ethiopia; 2 Department of Obstetrics and Gynecology, School of Medicine, College of Medicine and Health Sciences, University of Gondar, Gondar, Ethiopia; 3 Department of health informatics, Institute of Public Health, College of Medicine and Health Sciences, University of Gondar, Gondar, Ethiopia; 4 Department of Health Promotion and Health Behavior, Institute of Public Health, College of Medicine and Health Sciences, University of Gondar, Gondar, Ethiopia; Haramaya University Faculty of Health Sciences: Haramaya University College of Health and Medical Sciences, ETHIOPIA

## Abstract

**Background:**

Obstetric ultrasound is one of the most important advances in antenatal tests. Despite the importance of knowing the knowledge status of ultrasound and the category of attitude, there is no similar study done in Ethiopia. Thus, this study aimed to assess knowledge and attitude and associated factors toward ultrasound examination at the University of Gondar comprehensive specialized hospital, Northwest Ethiopia.

**Methods:**

Institutional-based cross-sectional study was conducted at the University of Gondar comprehensive specialized hospital, Northwest Ethiopia from May 15, 2022, to Jun 30, 2022. Data was collected and transferred from the Open data Kit server and analyzed using STATA version 14. The strength of the association, presented using an adjusted odds ratio with a 95% confidence interval and p-value ≤0.05, is considered to declare statistically significant variables.

**Results:**

A total of 422 pregnant women participated, with a response rate of 100%. Of the total pregnant women, only 39% of them are knowledgeable about obstetric ultrasound whereas 52% of them have a favorable attitude. Being a housewife, government employee, and private employee (AOR = 17, 95% CI = 2.12, 151), (AOR = 10, 95% CI = 1.2, 85) and (AOR = (13, 95% CI = 1.5, 115), respectively were associated positively with knowledge about ultrasound. However, residing in a rural (AOR = 0.07; 95%CI = 0.02, 0.21), never been pregnant before (AOR = 0.59 95% CI = 0.38, 0.94), and having information (AOR = 1.7, 95% CI = 1, 2.9) were negatively associated with knowledge about ultrasound. Whereas, attaining primary education (AOR = 2.61; 95%CI = 1.1, 6.4) was positively associated with favorable attitudes while living in rural areas was negatively associated with a favorable attitude (AOR = 0.42; 95%CI = 0.18, 0.97).

**Conclusion:**

In this study, knowledge and attitude about ultrasound among pregnant women in Gondar City are low. Residence, occupation, being pregnant before, and having information were significantly associated factors of knowledge. While residence and educational status of the respondents were significant factors of attitude. Therefore, health information about obstetric ultrasound shall be given to women who live in rural areas, women who are students, merchants and farmers, illiterate, and primigravida.

## Introduction

The systematic use and increasing dependence on sophisticated technologies continued to support a medicalized approach to childbirth. Globally, the medicalization of pregnancy has been enhanced by modern technological advances [1]. Ultrasound is one of the most widely used modern reproductive technologies in the world which delivers valuable diagnostic information [2,3]. It became an integral part of routine prenatal care for both high-risk and normal pregnancies around the world through advanced healthcare services [4].

Ultrasound is painless, gives immediate, wide-ranging results, and is widely regarded as safe [5,6]. Routine obstetric ultrasound is now recognized worldwide as one way of reducing maternal mortality. It is a critical and vital tool for the health care of women which helps healthcare providers to determine the fetal status and gestational age of the pregnancy [7–9]. It acts as a source of comfort regarding the well-being of the fetus and also a source of connection with her baby during the prenatal period [4]. Moreover, Pregnant women can view their first ultrasound as a tool that allows them to achieve various goals during pregnancy while the goals mainly concern meeting and having contact with the baby [10].

Ultrasound technology has reached almost universal coverage in developed countries, with particular emphasis on prenatal surveillance [11]. The advantages of diagnostic ultrasound in a context of lack of resources are well known and uncontested and it may bring real benefits to improve maternal and newborn health outcomes [3].

However, women often lack information about why an ultrasound is carried out and the technical limits of the procedure. So, the safety of diagnostic ultrasound during the prenatal period was unknown to most pregnant women [12–14]. Thus, mothers must be well informed about the safety and specific objectives of obstetric ultrasound and what it can and cannot accomplish [6].

A study showed that the common and significant factor that affects the use of obstetric examination was knowledge of ultrasound [15]. Another study also showed that in the low use of prenatal care, women do not have sufficient awareness of the importance of ultrasounds during pregnancy, so many congenital anomalies and obstetric complications happen to most parents unexpectedly [16]. Most women wanted ultrasounds even though many do not understand the procedure or purpose [17].

Its relevance is unquestionable when it is provided among prenatal programs, especially in a low-resource context. Thus, it is expected to become a standard procedure in developing countries but evidence showed that there are misconceptions and poor understanding of ultrasound in Ante Natal Care (ANC) and this was mainly be due to a lack of awareness about ultrasound. Furthermore, mothers must be well informed about the safety, purposes of obstetric ultrasound and what it can and cannot accomplish.

However, despite the importance of knowing their knowledge status towards ultrasound and the category of their attitude which have a big role in routine health care provision, to the best of our knowledge, there are a few studies in Ethiopia. So, this study provided important evidence inputs for addressing expectations as well as underestimation of pregnant women towards ultrasound usage that is observed in the routine ANC services. In addition, this study helps as a baseline to guide the policymakers in designing health messages which will persuade pregnant women to have a favorable attitude and to acquire good knowledge. Therefore, we aimed to assess the knowledge, attitude, and associated factors of pregnant women towards ultrasound examination at the University of Gondar comprehensive specialized hospital, Northwest Ethiopia.

## Methods and materials

### Study design and setting

Institutional-based cross-sectional study design was conducted in ANC at the University of Gondar Comprehensive Specialized Hospital, Northwest Ethiopia between May 15 to June 30, 2022. The hospital is found in the ancient and historic town of Gondar, northwest Ethiopia, 741 km from Addis Ababa which. consists of 4 operating rooms, 4 intensive care units, and 13 wards with more than 500 beds. The ANC is one of the departments which provide services to 50–70 pregnant women coming from Gondar town and the nearby districts per day [18,19].

### Population and eligibility criteria

All pregnant women who attended the ANC Gondar University comprehensive specialized hospital were the source population while pregnant women who are selected during the study period were the study population. Whereas, all pregnant women who were attending ANC follow-up at the time of data collection were eligible for the study but pregnant women who had psychiatric disorders and who were critically ill were not eligible for the study.

### Sample size determination and sampling procedure

The sample size was determined by using single population proportion formula;

$$n=(Za/2{)}^{2}p(1‐p)/d^{2}$$

Where; n–sample size,

Z is the Value of z statistic at 95% confidence interval = 1.96

P–The proportion of knowledge and attitude was taken at 50%, as there is no similar study in Ethiopia.

1-P = 1–0.5 = 0.5 and

d–Marginal error 5% = 0.05

So, the sample size for patient knowledge and attitude was calculated as follows:

n = (1.96)2 (0.5) (0.5) /0.05^2^ = 384

Therefore, by considering the 10% non-response rate, the final sample size for this specific study is 422.

A systematic random sampling technique was employed to recruit the respondents. First, the average number of pregnant women who attended ANC at the hospital every month was obtained by considering the previous months from the registration book at the clinic. Then, based on that estimation, on average 1700 pregnant women were estimated to visit ANC per month. The first women were selected by simple random sampling using a lottery method. A sampling fraction of 5 (= 1700/422) was calculated by dividing the average number of pregnant women receiving ANC in the previous months by the sample size then every fifth woman of the initial participants was included. However, the next participant was considered whenever the selected one did not fulfill the inclusion criteria or was unwilling to participate.

### Study variables

The dependent variables were knowledge and attitude about ultrasound examination. Whereas, the independent variables were sociodemographic and economic status, obstetric and modifiable factors, and health-related factors.

### Operational definition

#### Good knowledge

Good knowledge status of pregnant women that was taken when they scored median and above among fifteen knowledge questions.

#### Favorable attitude

This was taken when pregnant women scored median and above among eight attitude questions.

### Data collection procedures and data quality assurance

A pre-test was conducted in Felege Hiwot hospital to identify problems with questioners and amendments were made based on the pretest findings. An adapted standardized semi-structured questionnaire was utilized. A pre-tested semi-structured interviewer-administered questionnaire was translated into the Amharic language by language experts to collect the data. The data was collected using the Open Data Kit (ODK) application. The questionnaire consisted of four sections. The first section contains sociodemographic data. The second section focuses on clinical and modifying factors, the third section contains knowledge about ultrasound, and the fourth section contains attitude towards ultrasound examination. The data were collected by three first-year Master of science (M.Sc.) Midwifery students. They were trained for two days by the principal investigator on the study instrument, consent form, and data collection procedure. Master of public health (MPH) in maternal and child health experts supervised the procedure of the data collection.

To keep data quality the questionnaire (English version) was translated into Amharic and translated back to English. Two days of training were given to the data collectors on the objective, the relevance of the study, on the study instrument, consent form, data collection procedure, confidentiality of information and respondent’s right. To check content validity, the questionnaire was given to five maternal and public health experts with assistant professors and above qualifications.

Finally, the principal investigator incorporated the comments and prepared the final draft of the tool for data collection. Before the actual data collection, the questionnaire was prepared on the ODK server and linked to the ODK application. Then pre-tested at health centers (affiliation sites of this University hospital) on 5% of the final sample the principal investigator. The purpose of the pre-testing was to ensure whether respondents can understand the questions or not and to check the wording of the questions in a rational way to the respondents. After pre-testing, amendment such as many wording errors was modified and the English version was translated to Amharic. The supervisors made frequent checks on the data collection process to ensure the completeness & consistency of the gathered information and errors found during the process and corrected them accordingly. All data was stored in a locked filing cabinet accessible only to the researchers. Upon completion of the study, all information that matches up individual respondents with their answers was destroyed.

### Data analysis procedures

After collection, data is exported to STATA version 14 from the ODK server for further data management. Variable coding and transformations were done to make the data set ready for analysis. The descriptive analysis such as proportions, percentages, means, and measures of dispersion, tables, and graphs was done. Binary logistic regression modeling analysis was performed to assess the relationship between the knowledge and attitude of pregnant women and associated factors. The model fitness was evaluated through the fit index namely, Hosmer and Lemeshow. Missing data was removed before the analysis. A p-value was greater than 0.05 which indicated it was a good model fit. Whereas variables with p value less than 0.2 at the bivariable logistic regression model were entered into a final model of a binary logistic regression model. A p-value of less than 0.05 and a 95% confidence interval in the multivariable logistic regression model were used to declare statistically significant variables.

### Ethics approval and consent to participate

Ethical approval was obtained from the IRB (institutional review board) of the University of Gondar with reference number SOM/1420/2022. An official letter of permission was written by the Head of the School of Medicine of the University of Gondar specialized comprehensive hospital. Following an explanation of the purpose of the study verbal consent was obtained from participants. Also, affirmation was made that they are free to withdraw participation without any form of consequences and they were asked if they are willing to participate in the study. Data collection were started after receiving verbal consent from those who were willing to participate in the study. Participants gave informed consent to participate in the study before they were involved. Confidentiality of information and privacy of participants were assured for all the information provided, to preserve the confidentiality the data were not exposed to any third party except the principal investigator and advisors.

## Results

### Sociodemographic characteristics

A total of 422 pregnant women participated, with a response rate of 100%. The mean age was 27 ±5 years. Of the total respondents, three hundred forty-two (81%) were orthodox by religion. Sixty-two (15%) of the women couldn’t ‘t read and write. Two hundred thirty-three (55%) of them were housewives. Most of the respondents (89%) were urban dwellers (Table 1).

**Table 1:** Sociodemographic characteristics of pregnant women in the University of Gondar specialized hospital, Gondar, Northwest Ethiopia, 2022.

Variable	Description	Frequency/percent
Age	15–24	146(34.6%)
	25–34	231(54.7%)
	35–49	45(10.7%)
Religion	Orthodox	342(81%)
	Muslim	75(17.8%)
	Protestant	5(1.2%)
Residence	Urban	374(88.6%)
	Rural	48(11.4%)
Occupation	Housewife	233(55%)
	Government employee	75(18%)
	Private employee	101(24%)
	Other	13(3%)
Income	<2000 ETB	11(3%)
	2000–3999 ETB	82(19%)
	4000–5999 ETB	115(27%)
	6000–7999 ETB	93(22%)
	>8000 ETB	121 (29%)
Educational status of the respondent	Unable to read and write	62(15%)
	Can read and write	13(3.1%)
	Primary	104(24.7%)
	Secondary	123(29.2%)
	Diploma and above	120(28%)
Marital status	Single	9(2%)
	Married	413(98%)
Family size	< = 4	367 (87%)
	>5	55(13%)

*ETB-Ethiopian Birr.

### Obstetric factors

Most (96%) of the pregnant women were parous whereas nearly half of them had been pregnant at least once. Out of the two hundred four pregnant women, 13% of them had previous abortions while 5% of them had stillbirth (Table 2).

**Table 2:** Obstetric factors of pregnant women in the University of Gondar specialized hospital, Gondar, Northwest Ethiopia, 2022.

Variable	Description	Frequency /percent
Have you ever been pregnant before?	Yes	204(48%)
	No	218(52%)
Have you ever given birth?	Yes	195 (96%)
	No	9(4%)
Parity	Primiparous	92(47%)
	Multi-parous	100(51%)
	Grand- multiparous	3(2%)
Did you have any complications in your previous pregnancies?	Yes	11(5%)
	No	193(95%)
Do you have a chronic medical illness?	Yes	12(3%)
	No	410(97%)
Do you have a history of previous abortions?	Yes	26(13%)
	No	178(87%)
Do you have a history of stillbirth?	Yes	10(5%)
	No	194(95%)
Do you have a history of early neonatal death?	Yes	9(4%)
	No	195(96%)

### Knowledge about obstetric ultrasound

**Fig 1:**
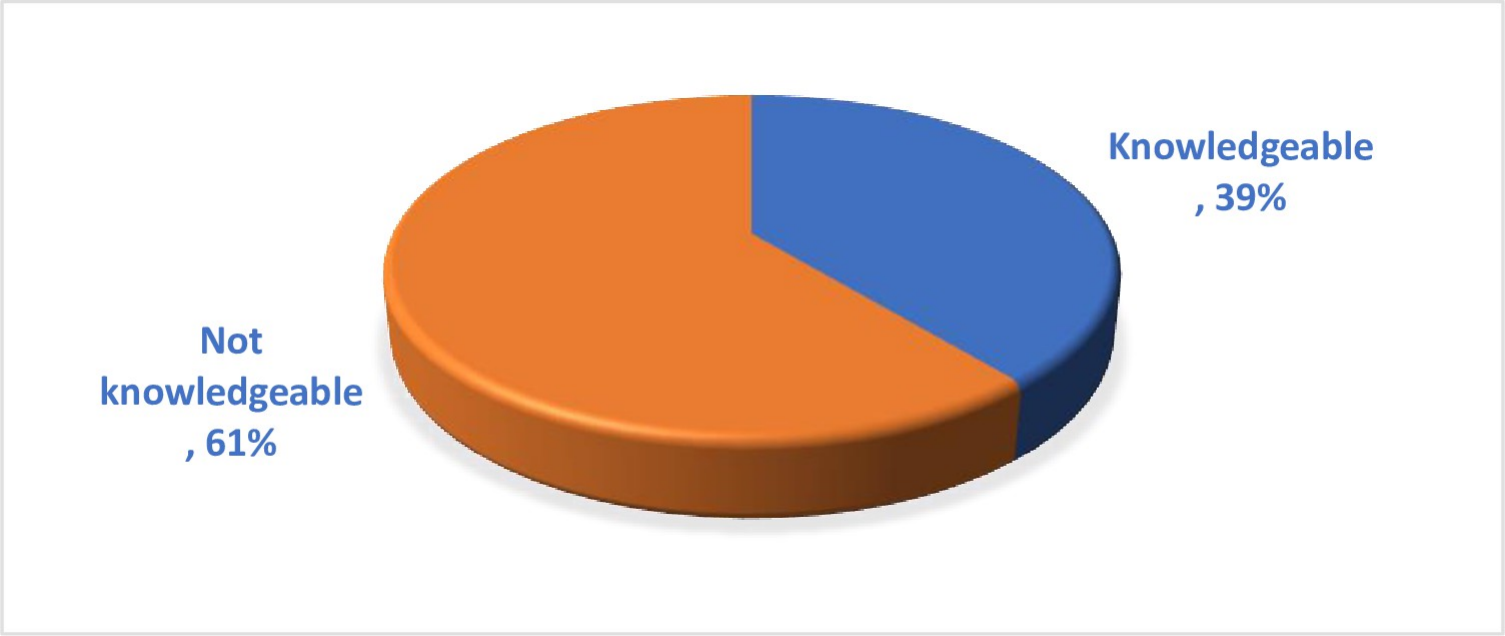
Knowledge about ultrasound among pregnant women in the University of Gondar specialized hospital, Gondar, Northwest Ethiopia, 2022.

Of the total of 422 pregnant women, 39% (165) of them are knowledgeable about obstetric ultrasound whereas 61% (257) are not-knowledgeable about obstetric ultrasound (Fig 1).

### Factors associated with Knowledge of obstetric ultrasound

In the bi-variable regression model residence, occupation, the way to confirm pregnancy, time of ANC visit, readiness for pregnancy, having husband support, being pregnant before, and having information were significantly associated with knowledge with P-value <0.2. In the multivariable model residence, occupation, being pregnant before, and having information were significant factors. Those who resided in rural decreased the odds of knowledge about ultrasound by 93% (AOR = 0.07; 95%CI = 0.02, 0.21). Moreover, those who are housewives, government employees, and private employees increased the odds of knowledge about ultrasound by 17,10 and 13 times, respectively (AOR = 17, 95% CI = 2.12, 151), (AOR = 10, 95% CI = 1.2, 85) and (AOR = (13, 95% CI = 1.5, 115). Those who have never been pregnant before decreased the odds of knowledge about ultrasound by 41% (AOR = 0.59 95% CI = 0.38, 0.94). Information about ultrasound increased the odds of knowledge about ultrasound by 1.7 times (AOR = 1.7, 95% CI = 1, 2.9) (Table 3).

**Table 3:** Factors affecting Knowledge of obstetric ultrasound among pregnant women in the University of Gondar specialized hospital, Gondar, Northwest Ethiopia, 2022.

Variable		COR (95% CI)	P-value	AOR (95% CI)
Residence	Rural	0.08(0.04, 0.19)	0.000	0.07(0.02, 0.21) *
	Urban	1		1
Occupation	Housewife	4.8(1.05, 22.3)	0.008	17(2.12, 151) *
	Government employed	2.3(0.47, 11.2)	0.033	10(1.2, 85) *
	Private employed	2.7 (0.56, 12.7)	0.02	13 (1.5, 115) *
	Others	1		1
How do you confirm the pregnancy?	Missed period	1		1
	Urine test kit	0.63 (0.42, 0.94)	0.69	0.9 (0.58, 1.44)
	Ultrasound	2.52(0.23, 28)	0.24	4.5(0.36, 3.56)
Time of ANC visit	≤12 weeks	1		1
	12–24 weeks	1.27(0.8, 2)	0.95	0.98(0.59, 1.64)
	>24 weeks	7(3.2, 15.4)	0.34	1.58(0.6, 4.13)
Readiness for pregnancy	Planned	1		1
	Unplanned	1.8(1, 3.2)	0.43	1.33(0.65, 2.69)
Do you have husband support?	Yes	1		1
	No	3.7(0.95, 14.6)	0.95	2.2(0.34, 0.42)
Have you been pregnant before?	Yes	1		1
	No	1.4(0.36, 5.37)	0.027	0.59(0.38, 0.94) *
Do you have information about ultrasound?	No	1		1
	Yes	2.4(1.49, 3.7)	0.047	1.7(1, 2.9) *

Others * for occupation = merchants, farmers and students.

### Attitude towards ultrasound

**Fig 2:**
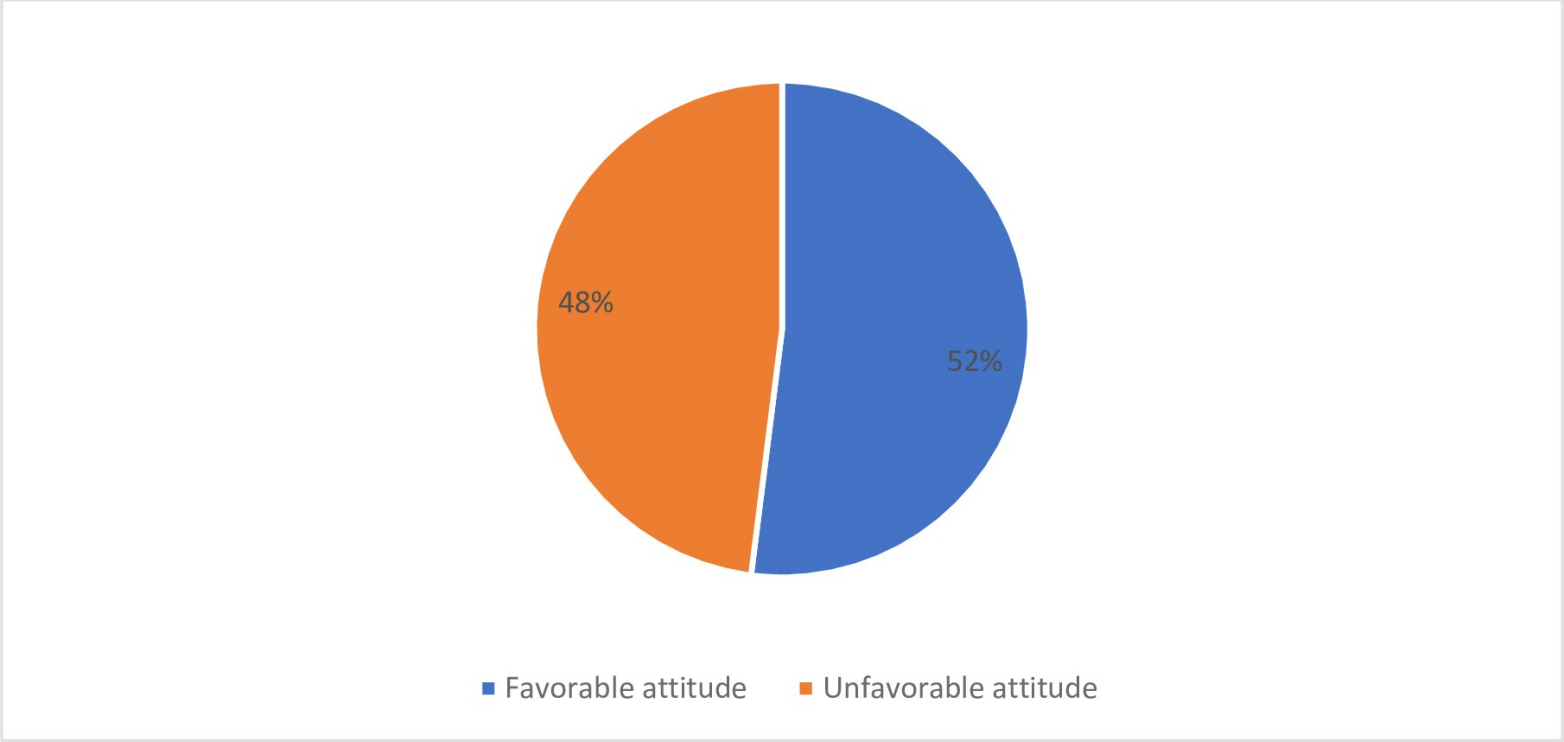
Attitude towards ultrasound among pregnant women in the University of Gondar specialized hospital, Gondar, Northwest Ethiopia, 2022.

Of the total of 422 pregnant women, 52% (220) of them have a favorable attitude toward obstetric ultrasound whereas 48% (202) have a non-favorable attitude towards obstetric ultrasound (Fig 2).

### Factors associated with attitude toward obstetric ultrasound

Age, educational status of the women, occupation, educational status of the husband, time of ANC visit, residence, family size, and being pregnant before were significant variables in the bivariable regression model with p-value <0.2. Among these, the educational status of the women and residents were significant variables in the multivariable regression model. Those who attained primary education increased the odds of a favorable attitude by 2.6 times (AOR = 2.61; 95%CI = 1.1, 6.4). But, those who lived in rural decreased the odds of the attitude towards obstetric ultrasound by 58% (AOR = 0.42; 95%CI = 0.18, 0.97) (Table 4).

**Table 4:** Factors affecting the attitude of obstetric ultrasound among pregnant women in the University of Gondar specialized hospital, Gondar, Northwest Ethiopia, 2022.

Variable		COR (95% CI)	P-value	AOR (95% CI)
Age	15–24	1		1
	25–34	0.96(0.63, 1.45)	0.41	1.24(0.74, 2.07)
	35–49	0.46(0.23, 0.9)	0.6	0.78(0.3, 2)
Educational status of the respondent	Can’t read and write	1		1
	Can read and write	3.35(0.98, 11.5)	0.61	1.45(0.34, 6.1)
	Primary	4.6(2.3, 9.2)	0.037	2.61(1.1, 6.4) *
	Secondary	4.5(2.3, 8.82)	0.195	1.97(0.7, 5.47)
	Diploma and above	2.7(1.37, 5.3)	0.73	1.21(0.4, 3.64)
Educational status of husband	Cannot read and write	1		1
	Can read and write	3.8(0.94, 15.4)	0.24	2.7(0.51, 14.3)
	Primary	2.7(1.34, 5.68)	0.74	1.2(0.45, 3)
	Secondary	4.1(1.9, 8.86)	0.4	1.6(.52, 4.9)
	Diploma and above	3.7(1.6, 6)	0.5	1.4(0.51, 4)
Time of ANC visit	≤12 weeks	1		1
	12–24 weeks	1.15(0.75,1.78)	0.51	1.2(0.74, 1.86)
	>24 weeks	0.44(0.22, 0.9)	0.8	0.89(0.37, 2.14)
Residence	Urban	1		1
	Rural	0.23(0.12,0.47)	0.043	0.42(0.18, 0.97) *
Family size	< = 4	1		1
	> = 5	0.57(0.32, 1)	0.09	1.03(0.48, 2.25)
Have you been pregnant before?	Yes	1		1
	No	1.5(1, 2.2)	0.43	0.67(0.67, 1.87)

## Discussion

The current study aimed to assess knowledge, attitude, and associated factors among pregnant women in ANC about obstetric ultrasound. This study revealed that only 39% of pregnant women had better knowledge about obstetric ultrasound with 95%, CI- 35% to 44%. Residence, occupation, being pregnant before, and having information were significantly associated with knowledge about ultrasound. Whereas 52% of them had a favorable attitude towards ultrasound with 95% CI-47% to 57%, the women’s residency and educational status were the significant factors.

The magnitude of knowledge of the current study was in line with a study done in Ethiopia [20]. However, it was much lower than a study done in Jeddah [9,16]. Whereas the magnitude of attitude of this study was lower than studies done in Jeddah and Ethiopia [9,20,21]. The discrepancies in the magnitude of knowledge and attitude of pregnant women towards obstetric ultrasound might be ascribed to differences in the study population, tools used to measure the outcome variables across the studies, and the sample size used.

In regards to the factors affecting knowledge of pregnant women’s, residence was one of the factors which affect the knowledge of women about obstetric ultrasound in which, those who lived in rural areas were less likely to have good knowledge than those who resided in urban areas. This finding was supported by a study done in Ethiopia [20].

Also, housewives, government employees, and private employees were more likely to had good knowledge than students, merchants, and farmers. This finding was consistent with a study conducted in Ethiopia, which found that government employees were significantly associated with knowledge [21]. Furthermore, this finding was confirmed by a study conducted in Jeddah, where employment was found to be associated with good ultrasonography knowledge as a result of having more opportunities to obtain information and share experiences with others [5,9]. This could be because such groups had more opportunities to receive information and acquire knowledge than others. In addition, those who were pregnant before were more likely to have better knowledge than those who had never been pregnant before. This result was supported by a study done in Jeddah and Uganda [6,9].

Furthermore, pregnant women with information were more likely to be knowledgeable. A study conducted in Germany demonstrated that information provision adds to higher knowledge, including for pregnant women with low levels of education [22]. Besides, A systematic review also found that unless women are given useful information regarding ultrasonography, they will not have up-to-date knowledge [12].

Those who resided in rural areas, on the other hand, were less likely to have favorable attitudes than those who resided in urban areas. This finding matches up to one seen in Cameroon [7]. One possible explanation is that those who resided in urban had better access to information and may gain knowledge more about obstetric ultrasound, which helps them to had a positive attitude. Likewise, those who completed primary school had a more positive attitude than those who couldn’t read or write. This finding was similar to that of a Ugandan study [6].

### Strength and limitation of the study

The current study studied one of the most important advances of antenatal tests and obstetric emergency care which is recognized as one way of reducing maternal mortality throughout the world. The limitation of this study is, it is impossible to make causal inferences due to the cross-sectional nature of the study. The sample size of the current study might not be enough to generalize its findings. The other limitation is recall bias which might under/overestimate the magnitude of the outcomes. While the study was designed as an institution-based cross-sectional study, it did not include women who received antenatal care at a private clinic.

## Conclusion and recommendation

In this study, knowledge and attitude about ultrasound among pregnant women in Gondar City were low. Residence, occupation, being pregnant before, and having information were significant factors of knowledge. Whereas residence and educational status of the respondents were significant factors of attitude. Therefore, health information about obstetric ultrasound should be given to women who live in rural areas, women who are students, merchants, farmers, illiterate, and primigravida.

## Supporting information

S1 Checklist STROBE statement—Checklist of items that should be included in reports of observational studies. (DOCX)

## Abbreviations

ANC: Ante Natal Care
AOR: Adjusted Odds Ratio
COR: Crude Odd ratios
DM: Diabetes Miletus
HTN: Hypertension
ODK: Open Data Kit
M. Sc: Master of Science
MPH: Master of Public Health
